# S10. ASTROGLIAL PATHOLOGY IN SCHIZOPHRENIA: A META-ANALYSIS OF MRS STUDIES OF ANTERIOR CINGULATE MYOINOSITOL

**DOI:** 10.1093/schbul/sby018.797

**Published:** 2018-04-01

**Authors:** Avyarthana Dey, Priyadharshini Sabesan, Jean Theberge, Joaquim Radua, Lena Palaniyappan

**Affiliations:** 1 University of Western Ontario; 2 London Health Sciences Center; 3 St. Joseph’s Health Care London; 4 IoPPN, King’s College London

## Abstract

**Background:**

Several lines of evidence support a role for astroglial pathology in schizophrenia. 1H-MRS does not specifically differentiate between brain cell types; nevertheless, given that myo-inositol (mIns) is particularly abundant in astroglia rather than neuron and microglial cells, it can be considered an astroglial marker. mIns levels in the brain can decrease after brain injury with an efflux of mIns from astrocytes occurring as an osmoregulatory response. Many small sized studies have reported on mIns concentration in schizophrenia, but to date these have not been pooled to estimate a collective effect size. Examining the state of mIns deficit is a critical step to delineate the role of astroglial cells in schizophrenia. We conducted a meta-analysis to investigate the aberrations in myo-inositol levels in the ACC of patients with schizophrenia and measured using magnetic resonance spectroscopy (MRS).

**Methods:**

Medline, Google Scholar, Ovid Online and EMBASE databases were searched for studies published until September 2017. Search terms included full forms and variations of magnetic resonance spectroscopy, MRS, schizophrenia, psychosis, myo-inositol, inositol, Ins, mI, mIns. We included all 1H-MRS studies reporting mIns values for patients satisfying DSM or ICD based criteria for a primary psychotic disorder (SCZ) in comparison to a healthy controls (HC) group. We screened all identified abstracts, filtered studies that did not satisfy inclusion criteria, hand-searched references and contacted experts to locate further studies. 9 studies were identified that included 223 patients in SCZ group and 231 HCs. We excluded studies that reported only on comorbid illnesses, did not compare patients and HCs, or failed to report data required to construct effect size metrics. A random-effects and fixed-effects, inverse-weighted variance model was used to calculate the pooled effect size. Mean values were extracted and verified independently and effect sizes were computed based on Excel Macro produced by Major Depressive Disorder Neuroimaging Database (MaND) investigators.

**Results:**

Contrary to our expectations, in SCZ, there were no significant differences in ACC mIns in patients compared to HC (RFX=0.359, p= 0.057; 95% CI, -0.728 to 0.011; heterogeneity p = 0.0004). In the SCZ group, the mean effect size (Cohen’s d) was d= 0.39, indicating a medium sized difference. There were several methodological issues in the reported studies. Notably, most studies reported on mIns spectrum only when seeking differences in other metabolites; voxel placements were not standardized across the published studies; majority of patients were medicated, in various stages of illness. There was no statistical evidence for a publication bias (p=0.8).

**Discussion:**

There is a medium effect-size, albeit statistically insignificant, reduction in the concentration of mIns in the anterior cingulate cortex in patients with schizophrenia. Given that mIns is the most readily accessible cortical marker in vivo for astroglial activity, it may be feasible to use MRS to stratify patients with astroglial abnormality from those without such an abnormality in schizophrenia.

